# Orbital Metastasis as the Initial Manifestation of Lung Adenocarcinoma: 18F-FDG PET-CT Findings

**DOI:** 10.1055/s-0045-1808247

**Published:** 2025-04-25

**Authors:** Oueriagli Nabih Salah, Aboussabr Meryem, Ait Sahel Omar, Doudouh Abderrahim

**Affiliations:** 1 Department of Nuclear Medicine, Med V Military Teaching Hospital, BP, Rabat, Morocco

**Keywords:** 18F-FDG, lung adenocarcinoma, orbital metastasis, PET/CT

## Abstract

Orbital metastases from malignant tumors are uncommon. In 25% of cases, they are the first sign of an undiagnosed cancer, and they account for approximately 7 to 12% of lung cancer cases. A lack of awareness about this condition can lead to misdiagnosis, distinguishing malignant from benign lesions. We present the case of a 65-year-old patient with orbital metastasis from lung cancer. 18F-fluorodeoxyglucose (18F-FDG) positron emission tomography/computed tomography (PET/CT) was crucial in diagnosing this, revealing hypermetabolism in the left lung mass as well as intense uptake in the right retro-orbital region, which was confirmed as orbital metastasis through cerebro-orbital magnetic resonance imaging. For 2 months, our patient had right eye pain and decreased visual acuity and no attention was paid to these symptoms. Through this clinical case, the authors highlight the utility of 18F-FDG PET/CT in the diagnosis of primary malignancy in lung cancer patients, who presented with orbital metastasis as the first sign.

## Introduction

Orbital metastases are relatively uncommon, with breast and lung cancers being the most common primary tumors, accounting for 0.7 to 12% of cases. 1 2 The choroid is the ocular tissue most frequently affected by metastatic disease, followed by the iris and ciliary body. 3 4 In 25% of cases, orbital metastases represent the first manifestation of an unknown primary carcinoma (UPC), where 18F-fluorodeoxyglucose (18F-FDG) positron emission tomography/computed tomography (PET/CT) plays a critical role. We present a case of a patient with orbital metastasis incidentally detected by an 18F-FDG PET/CT with lung cancer.

## Case Report

A 65-year-old male, a chronic smoker, presented with right eye pain and decreased visual acuity. Initially, this symptom was overlooked. Two months later, the patient developed a chronic cough with hemoptysis, diffuse bone pain, and weight loss. A whole-body CT scan revealed a left pulmonary mass measuring 32 mm × 25 mm, along with suspicious mediastinal lymph nodes.

**Fig. 1:**
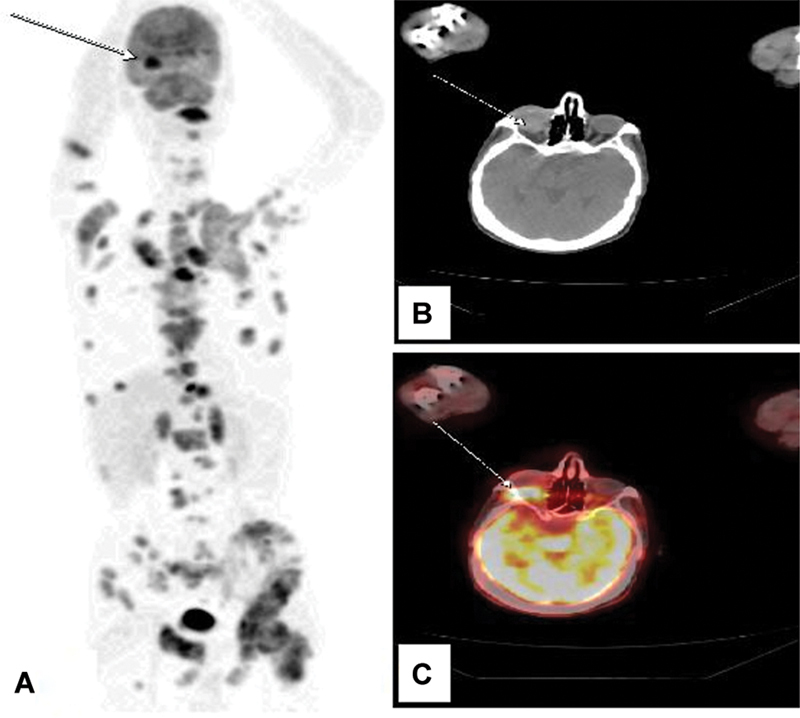
( **A** ) Maximum intensity projection (MIP) of our patient showing normal and pathological uptakes. ( **B** ) Computed tomography (CT) in axial section of the orbital region showing retro-orbital mass in the right eye (Wight arrow). ( **C** ) Fusion image in axial section of the orbital region showing a suspect retro-orbital uptake in the right eye related to a retro-orbital metastasis (Wight arrow).

A biopsy of the lung mass confirmed invasive, moderately differentiated adenocarcinoma, with immunohistochemical markers (TTF1 + ) and an epidermal growth factor receptor (EGFR) mutation (L858R), indicating a bronchopulmonary origin. An 18F-FDG PET/CT scan showed intense uptake in the left lung mass (maximum standardized uptake value [SUVmax] = 6.5), multiple FDG-avid lymph nodes, and lesions in the liver, adrenal glands, and bones ( Fig. 1 A). Notably, there was also intense and suspicious retro-orbital uptake in the right eye (SUVmax = 10.5) ( Fig. 1 B and C).

**Fig. 2:**
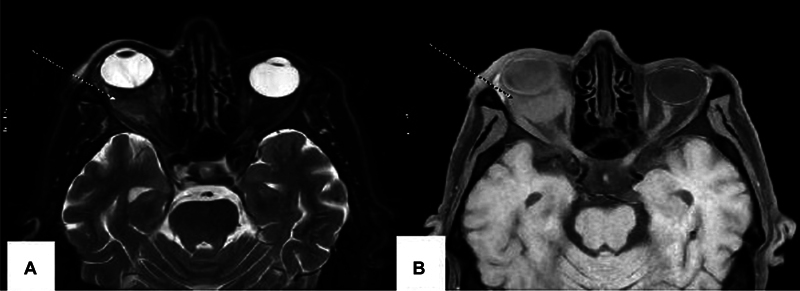
Magnetic resonance imaging (MRI) of the orbital region showing a 30 × 30 × 22 mm right retro-orbital mass isointense on T1 ( **A** ) and hyperintense on T2 with diffusion restriction ( **B** ) (Wight arrows), involving the optic nerve, intraconal fat, ocular rectus muscles, and the sphenoid wing.

Ophthalmological examination revealed retinal detachment, suggestive of a tumoral origin. Magnetic resonance imaging showed a 30 × 30 × 22 mm retro-orbital mass (isointense on T1, hyperintense on T2, with diffusion restriction), involving the optic nerve, intraconal fat, ocular rectus muscles, and the sphenoid wing ( Fig. 2 A and B), as well as a lesion in the left cerebellum.

The patient was treated with targeted therapy (erlotinib 150 mg daily) and radiotherapy for the orbital and cerebral metastases, achieving favorable outcomes.

## Discussion

Orbital metastases are rare, comprising 10% of orbital tumors and 3 to 7% of orbital lesions. 5 They typically affect the posterior choroid, with only 5 to 11% involving the ciliary body or iris. 5 Approximately in one-third of cases the primary malignancy is unknown. 6 Common primary sources include breast (39–48%), prostate, melanoma (12%), lung (8%), and kidney (7–11%). 7 Metastatic adenocarcinoma is considered as the predominant histological type. 8

Orbital symptoms such as proptosis, pain, and chemosis may indicate an undiagnosed primary cancer in 15% of cases. 9 Ocular metastases are often associated with widespread disease, with average survival ranging from 7.5 to 13 months after diagnosis. 10 For symptomatic patients, ophthalmological screening is recommended. 11

18F-FDG PET/CT is highly sensitive in detecting UPCs and staging disseminated disease. In one study, PET/CT identified primary tumors in 39.5% of cases, with lung cancer being the most common (50%). Sensitivity, specificity, and accuracy were reported as 87, 88, and 87.5%, respectively. 12 13

The treatment for orbital metastases is primarily palliative. Radiotherapy achieves a 79% response rate and preserves vision in 80% of cases. 14 Targeted therapies, such as erlotinib for EGFR-mutated adenocarcinoma, have shown to improve outcomes. Fractionated orbital radiotherapy (30–40 Gy) helps reduce complications like damage to the lacrimal apparatus. 15 The prognosis remains poor, with a 54% mortality rate within 1 year of ocular metastasis diagnosis. 10

## Conclusion

Orbital metastasis can be the first sign of carcinoma in up to 25% of cases. Adenocarcinoma represents 92% of lung cancer-related orbital metastases. Early suspicion and the use of multimodal imaging, such as 18F-FDG PET/CT, are essential for prompt diagnosis and intervention to preserve vision and quality of life. This case highlights the importance of PET/CT in detecting primary malignancies in patients presenting with orbital metastasis.
